# Type-I Interferon is Critical for FasL Expression on Lung Cells to Determine the Severity of Influenza

**DOI:** 10.1371/journal.pone.0055321

**Published:** 2013-02-08

**Authors:** Daisuke Fujikura, Satoko Chiba, Daisuke Muramatsu, Mika Kazumata, Yosuke Nakayama, Taro Kawai, Shizuo Akira, Hiroshi Kida, Tadaaki Miyazaki

**Affiliations:** 1 Department of Bioresources, Hokkaido University Research Center for Zoonosis Control, Kita-ku, Sapporo, Hokkaido, Japan; 2 Japan Science and Technology Agency, Innovation Plaza Hokkaido, Kita-ku, Sapporo, Hokkaido, 060-0819, Japan; 3 Aureo Science Co., Ltd., Kita-ku, Sapporo, Hokkaido, Japan; 4 Laboratory of Host Defense, World Premier International Research Center, Immunology Frontier Research Center, Osaka University, Suita, Osaka, Japan; 5 Department of Host Defense, Research Institute for Microbial Diseases, Osaka University, Suita, Osaka, Japan; 6 Hokkaido University Research Center for Zoonosis Control, Kita-ku, Sapporo, Hokkaido, Japan; 7 Graduate School of Veterinary Medicine, Hokkaido University, Kita-ku, Sapporo, Hokkaido, Japan; 8 Office International des Epizooties (OIE) Reference Laboratory for Highly Pathogenic Avian Influenza, Sapporo, Hokkaido, Japan; George Mason University, United States of America

## Abstract

Infection of influenza A virus in mammals induces hyper lung pneumonia, which often causes lethal diseases. FasL is a specific ligand of Fas, which is a type-I transmembrane protein to induce cell death. Previously, it has been reported that the hyper induction of gene expression associated with Fas signal is observed in lethal influenza A virus infection. More importantly, it was also reported that functional mutation of the *FasL* gene protects the host against influenza A virus infection. These observations suggest that induction of FasL signal is functionally associated with the severity of influenza. However, regulation of the induction of FasL or Fas by influenza A virus infection is still unknown. Here, we demonstrated that FasL is induced after the viral infection, and inhibition of the Fas/FasL signal by treatment with a recombinant decoy receptor for FasL (Fas-Fc) increases the survival rate of mice after lethal infection of influenza A virus as well as functional mutation of the *FasL* gene in gld/gld mice. In addition, the induction level of *FasL* gene expression in the lung was correlated with the severity of influenza. We also showed that a variety of types of cells in the lung express FasL after the viral infection. Furthermore, type-I interferon induced by the viral infection was shown to be critical for induction of FasL protein expression in the lung. These findings suggested that expression of FasL protein induced by type-I IFN on the lung cell surface is critical to determine the severity of influenza.

## Introduction

Influenza A virus infection causes acute respiratory inflammation and leads to lethal diseases including hyper lung pneumonia. It is known that influenza A viruses initially infect air-way epithelial cells and induce hyper production of several cytokines or chemokines. These cellular products induce anti-viral effects including direct inhibition of viral replication or recruitment and activation of several immune cells, such as macrophages, neutrophils or lymphocytes to eliminate the viruses or virus-infected cells [1]. FasL is a specific ligand of Fas, which is a type-I trans-membrane protein to induce cell death [2]. Functional mutation of the *FasL* or *Fas* gene causes abnormal proliferation of peripheral lymphocytes [3]. In immunological events, it is proposed that FasL protein expressed on killer T or natural killer cells plays a role in effector function for eliminating virus-infected cells and at a late phase after the infection, FasL/Fas signaling is essential for the suicide mechanism for activated peripheral lymphocytes to terminate inflammation [2]. Recently, it has been shown by DNA microarray analysis using mice infected with the highly pathogenic H1N1 influenza A virus (r1918 strain) comparing with the non-lethal virus (T×91 strain) that induction of the expression of FasL/Fas signal related genes in the lung is associated with the mortality of mammalians after the infection [4]. It is also reported that influenza A virus infection induces cell death of the infected cells by Fas-dependent apoptosis [5]. More importantly, it has been demonstrated that *FasL* gene functionally mutated congenic B6Smn.C3-Tnfsf6^gld^/J mice are more resistant to lethal influenza virus infection than C57Bl/6J mice [6]. Other studies demonstrated that activation of Fas signaling mediated by the administration of recombinant FasL protein or an anti-Fas agonistic antibody causes acute lung inflammation [7]–[9]. These findings suggested that the activation of FasL/Fas signaling in the lung is associated with the severity of the illness in lethal influenza virus infection.

Type-I interferon is known as an anti-viral cytokine, which induces the expression of several intracellular proteins including OAS, RNase L and Mx proteins resulting in the reduction of virus production [10]. Production of type-I IFN is regulated by receptor proteins directly recognizing virus RNA, such as Toll like receptors (TLRs) and retinoic acid-inducible gene-I (RIG-I) like proteins in virus-infected cells [11]–[13]. Recently, other functions of type-I IFN have been reported (reviewed in [14]). Previously, type-I IFN was shown to augment T-cell death induced in the activation states by up-regulating the expression of FasL and Fas [15]. More recently, it has been proposed that type-I IFN should contribute to the depletion of CD4 T cells in an HIV infection [16]. These findings suggested that type-I IFN regulates T cell proliferation in the viral infection.

In the present study, we demonstrate that in the lung of mice lethally infected with influenza A virus, FasL expression is induced more rapidly and abundantly than that in the lung of mice non-lethally infected with the virus. In addition, prevention for FasL/Fas interaction by administration of antagonist or functional mutation on *FasL* gene protects mice against lethal viral infection and prevents reduction of CD3 (+) cell population, which mediated by lethal infection with the virus in the lung. It is also demonstrated that abnormal production of type-I IFN is essential for highly induction of FasL expression on cell surface in the lung of mice lethally infected with influenza virus. These findings suggested that abnormal production of type-I IFN which causes highly induction of FasL expression on cell surface determines the severity of illness by influenza A virus infection.

## Materials and Methods

### Mice

C57BL/6 background gld/gld (B6-gld/gld) mice which have a functional mutation on *FasL gene*, preventing cell surface expression of the gene product [17], [18] and control C57BL/6 (B6) mice were purchased from SLC Inc. (Shizuoka, Japan). C57BL/6 background IFNR deficient mice (B6-IFNR-KO mice), in which the IFN alpha/beta receptor gene is specifically targeted, were described in [19]. These mice were housed in specific pathogen free condition. We performed animal care and experiments in accordance with guidelines and approval of the Animal Care and Use Committee of Hokkaido University.

### Infection

Mouse-adapted inﬂuenza virus A/PR/8 was prepared as previously described [20]. 6–8 week old male mice were lightly anesthesia with isoflurane (Dainippon Pharmaceutical, Osaka, Japan), and intranasally infected with a dose of 1×10^5^ or 1×10^2^ pfu/head of PR/8 virus in 50 ul of PBS. Body weights of the mice were monitored daily and assessed for visual signs of clinical disease including inactivity, ruffled fur, laboured respiration and huddling behaviour. Mice that lost ≥25% of their original body weight and/or displayed evidence of pneumonia were euthanized by overdose of inhalant anesthetic.

These experiments were conducted under animal BSL2 condition.

### RNA Preparation and Quantitative Real Time PCR

Total RNA was isolated from lung of mice using Trizol reagent (Invitrogen, San Diego, CA). Total RNA (5 µg) was reverse*-*transcribed using *ReverTra Ace* (Toyobo Co. Ltd., Osaka, Japan) with random primer and oligo-dT primer. Real-time PCR was performed with MX3000P instrument (Stratagene, Cedar Creek, TX) using SYBR® Premix Ex Taq™ II (Takara bio, Otsu, Japan). The primer sequences for target genes were as follows:

For mouse *FasL*

forward, 5′-AAGAAGGACCACAACACAAATCTG-3′,

reverse, 5′-CCCTGTTAAATGGGCCACACT-3′,

For mouse *Fas*

forward, 5′-CTGCGATGAAGAGCATGGTTT-3′,

reverse, 5′-CCATAGGCGATTTCTGGGAC-3′,

For mouse *GAPDH*

forward, 5′-AAGGGCTCATGACCACAGTC-3′,

reverse, 5′-GGATGCAGGGATGATGTTCT-3′.

Cycling conditions were used as: 95°C for 10 sec to activate DNA polymerase, followed by 40 cycles of 95°C for 5 seconds and 60°C for 30 seconds. Specificity of amplification products was confirmed by melting curve analysis. Each sample was assayed in triplicate in independent reactions.

### Plaque Assay

Madin-Darby canine kidney cells in a semiconfluent monolayer on 12 well culture plates were infected for 1 h at room temperature with serial 10-fold dilution of supernatant from lung homogenate in serum-free MEM medium. Unbound viruses were removed by washing the cells with MEM. Cells were then overlaid with MEM containing 0.7% Bacto-agar (Difco, Sparks, MD) in the presence of trypsin (5 µg/ml). At 48 hr after incubation at 35°C, the number of plaques was counted.

### Production of Recombinant Fas-Fc Protein

The DNA fragments coding sequences for extracellular region of mouse Fas and the Fc region of human IgG1 (hinge, CH2 and CH3 domains, containing point mutations at the position E233P/L234A/L235A for preventing its binding activity for Fc receptor [21]) were obtained by PCR and were cloned into the mammalian expression vector pcDNA3.1(+) (Invitrogen), as designated pcDNA3.1/mFas-hFc.

Human embryonic kidney 293 T cells were transfected with the plasmid pcDNA3.1/mFas-hFc with the selection plasmid containing puromycin-resistant gene (pGL4.1), and selected by puromycin (3 µg/ml). The selected cells were re-seeded in poly-L-lysine coated T175 flask. After overnight incubation, the cells were 4 times washed with PBS and re-cultured in serum free medium (CD293 medium, Invitrogen) containing Glutamax (Invitrogen). After 7 days of incubation, culture medium was collected and the secreted fusion proteins were purified by using recombinant protein A affinity column (HiTrap™ rProtein A FF, GE Healthcare, Uppsala, Sweden). After dialysis against PBS and concentration, the proteins were stored at −80°C until the administration. Purity of the recombinant protein was determined by SDS-PAGE (>90%).

### Lung Cell Preparation and Flowcytometry

Mice were sacrificed by cervical dislocation and lung was isolated from the mice and collected into C tube containing 4.9 ml HEPES buffer (ph 7.4) with 2 µg/ml collagenase-D and 40 U/ml Dnase I. The tissue was homogenized by gentleMACS™ Dissociator (Miltenyi Biotech, Bergisch Gladbach, Germany ) and then incubated at 37°C, 30 min with gentle rotation. After incubation, tissue was re-homogenized and filtrated by ø70 µm membrane filter. After centrifugation (300×g, 10 min, 4°C), cells were suspended in MACS buffer (PBS (pH7.2) containing 0.5% BSA and 2 mM EDTA). Viability of the cells after the preparation was >80% by 7-AAD staining.

For flowcytometrical analysis, cells (1×10^6^ cells) were preincubated with anti*-*CD16/CD32 mAb (clone 2.4G2) to avoid non-specific binding of antibodies to FcγR and then incubated with direct labeled mAbs at 4°C. After washing with MACS buffer twice, the cells were stained with 7-AAD for detecting dead cells in samples and fluorescent activities of the samples were analyzed by a FACS Canto (BD Biosciences, San Jose, CA). Fluorescent filter for phycoerythrin was used as depletion of auto-fluorescent cells in samples. Allophycocyanin (APC) or fluorescein (FITC)-conjugated anti-CD3 (500-A2), anti-CD4 (YTS191.1), anti-CD8 (KT15), APC-streptavidin and 7-AAD staining solution were purchased from Beckman coulter company (Fullerton, CA). FITC-anti-CD45R/B220 (RA3-6B2), FITC-anti-Ly6G (1A8), Alexa488-anti-podoplanin/gp36 (8.1.1), FITC-anti-CD11c (N418) and PE/Cy7-anti-F4/80 (BM8) were from Biolegend company (San Diego, CA). Biotin-anti-CD95L (MFL3) was from eBioscience company. Purified anti-CD16/32 (2.4G2), biotin-anti-CD95 (Jo2), FITC-anti-CD-74 (In-1) and rat or hamster IgG isotype control were from BD Biosciences company (Oxford, UK).

### Assessment of IFN- ß Concentration in Bronchoalveolar Lavage Fluid (BALF)

At the indicated day after infection, mice were sacrificed by cervical dislocation, and the lungs of mice were lavaged with 500 µl of phosphate-buffered saline (PBS, without calcium and magnesium, pH 7.4, and prewarmed at 37°C). The ﬂuid was infused, recovered and placed immediately on ice. The BALF was centrifuged at 300×g for 10 min at 4°C, and the cell-free supernatant was stored at −80°C. The amount of IFN-β was assessed by mouse IFN-beta ELISA kit (R&D systems, Abingdon, UK).

## Results

### Prevention of the Interaction of Fas with FasL Reduces the Mortality of Mice Infected with Influenza A Virus

**Figure 1:**
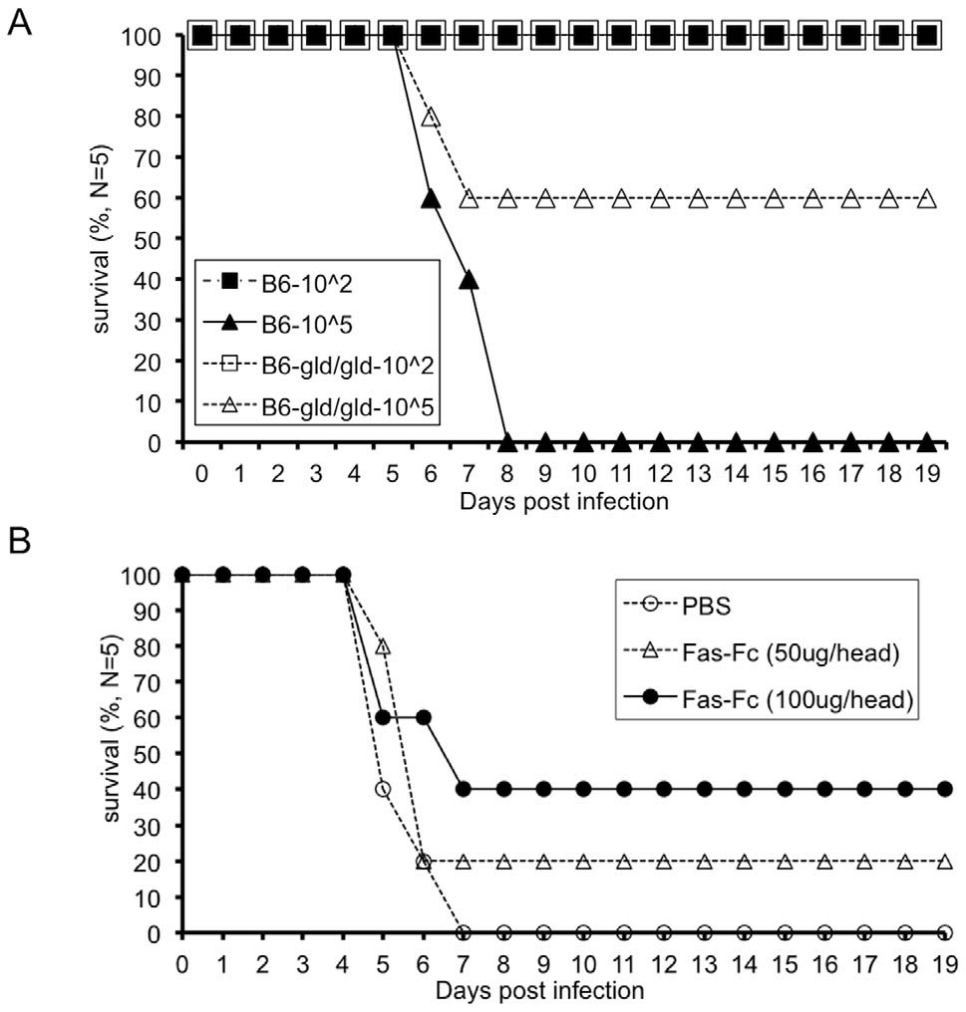
The interaction of FasL with Fas decreases the survival rate of mice with an influenza virus infection. (**A**) Control B6 (closed) mice or B6-gld/gld (open) mice were intranasally infected with 10^2^ (square) or 10^5^ (triangle) pfu/head of the PR/8 virus. Percentage of the mice that survived is shown for each group of 5 mice. (**B**) B6 mice were infected with 10^5^ pfu/head of the PR/8 virus and treated with Fas-Fc chimeric protein at 50 µg/head (1shot/2days until 4DPI, open triangle) or 100 µg/head (daily until 4DPI, closed circle) or PBS control (daily until 4DPI, open circle). Survival rate of these mice is shown (N = 5).

To evaluate the functional significance of FasL concerning to the severity of illness induced by influenza A virus infection in B6mice, the survival rates of B6-gld/gld mice were compared with that of control B6 mice after infection with titers (10^5^ or 10^2^ pfu/head) of PR/8 virus. In control B6 mice intranasally (i.n.) infected with 10^5^ but not 10^2^ pfu/head of the virus, a reduction of survival rate was highly observed at 6 days post infection (DPI) and all these mice were dead at 8DPI. In contrast, 60% of the B6-gld/gld mice infected with 10^5^ pfu/head of the virus survived until 19 days after the infection (Fig. 1A). In addition, treatment with recombinant decoy receptor for FasL, which consisted of the extracellular region of mouse Fas fused with the Fc region of human IgG (Fas-Fc) protected B6 mice against lethal infection of PR/8 virus in a dose dependent manner (Fig. 1B). These findings suggested that the signal mediated by the interaction of FasL with Fas is critical to determine the survival rate of mice lethally infected with the PR/8 virus.

### Expression of *FasL* but not *Fas* Gene in the Lung Correlates with the Severity of Illness in Mice after Influenza A Virus Infection

**Figure 2:**
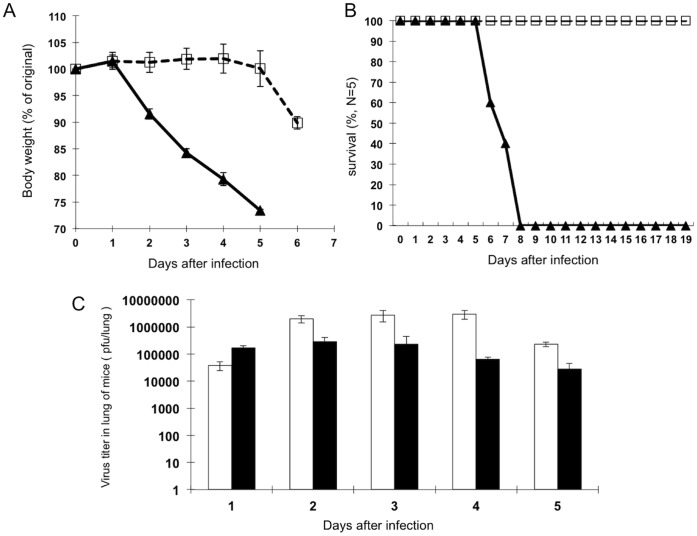
Virus titer in the lungs of mice does not correlate with the severity of the influenza infection. B6 mice (5 mice/group) were infected with 10^5^ (closed triangle) or 10^2^ (open square) pfu/head of the PR/8 virus. Changes in body weight (**A**) or survival rate (**B**) of these mice were shown. At the indicated days after the infection, the virus titer in the lungs of mice infected with 10^5^ (closed) or 10^2^ (open) pfu/head of PR/8 virus was assessed by plaque assay (C, N = 3/each time point).

It is known that the initial infected titer of the virus regulates the severity of illness such as loss of body weight and death of mice after influenza A virus infection. In B6 mice, infection with a high titer (10^5^ pfu/head i.n.) of PR/8 virus dramatically decreased the body weight of mice at 2∼5DPI (Fig. 2A, closed triangle) and all mice were dead at 8 DPI (Fig. 2B, closed triangle). On the contrary, in the mice infected with a low titer of the virus (10^2^ pfu/head, i.n.), reduction of body weight was slightly observed at 5∼6 DPI, and all these mice survived until 19 DPI (Fig. 2A and B, open square). By plaque assay, at 1DPI, the virus titer in the lungs of mice infected with a high titer was shown to be significantly higher, but was lower compared to that with a low titer of the virus after 2DPI (Fig. 2C). As shown in a previous report [6], these findings suggested that the initial infected but not propagated virus titer in the lungs of mice correlate with the severity of symptoms or mortality of mice after influenza A virus infection.

**Figure 3:**
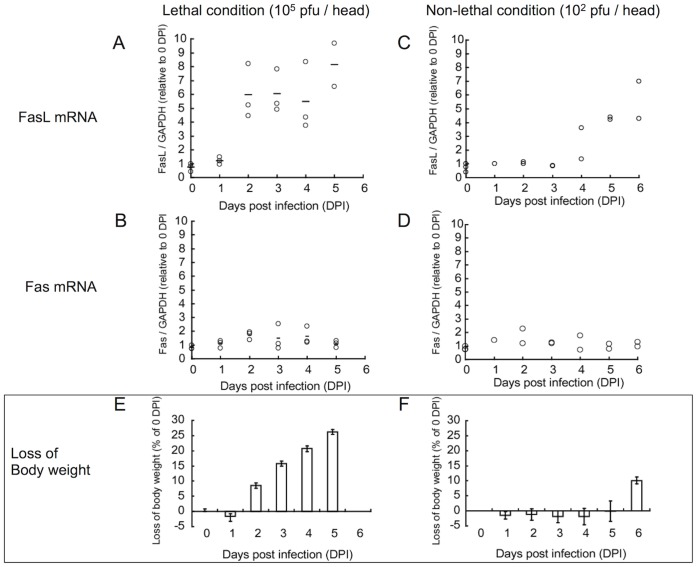
Induction of FasL gene in the lungs of mice infected with the PR/8 virus. B6 mice were infected with the PR/8 virus at the indicated virus titer. These mice were sacrificed at the indicated day, and mRNA expression of *FasL* (**A** and **C**) or *Fas* (**B** and **D**) in the lungs of these mice were assessed as described in *Materials and Methods*. The mean value of *GAPDH* was used for the internal control. Changes in body weight of mice infected with a lethal (**E**) or a non-lethal (**F**) virus titer were shown as percentage of the reduction compared with the original body weight (N = 3/each group).

To clarify the correlation of the function of *Fas* or *FasL* gene with the severity of illness in this model, their expression in the lungs of these mice were assessed by quantitative real time PCR (QPCR) methods using specific primer sets for these genes. In a high virus titer infection (lethal condition, 10^5^ pfu/head i.n.), a very high expression of *FasL* gene was observed at 2DPI and this expression level was sustained until the mice died (Fig. 3A). Compared with *FasL* gene, expression level of *Fas* gene was slightly increased during the infection (Fig. 3B). In a low virus titer infection (non-lethal condition, 10^2^ pfu/head i.n.), induction of *FasL* gene expression was observed after 4DPI (Fig. 3C) and *Fas* gene expression was not changed (Fig. 3D). It has been demonstrated that the induction level of *FasL* gene expression is correlated with body weight loss in both lethal and non-lethal conditions (compared with Fig. 3A versus 3E, and Fig. 3C versus 3F). These findings suggested that the gene expression level of *FasL* but not *Fas* is important to determine the severity of illness in mice infected with PR/8 virus.

### Type-I Interferon Signal is Essential for the Induction of FasL Protein Expression in the Lungs of Mice

Regarding the mechanism for regulating FasL protein induction by virus infection, there are two possibilities. One is that a virus component, such as viral RNA or protein should directly activate an intracellular signaling, which induces FasL expression. The other is that some cytokines including type-I interferon (IFN), which is produced by virus infected cells, should induce FasL expression. To clarify these possibilities, we assessed the effect of shut down on a type-I interferon (IFN) signal on FasL expression induced with the viral infection.

**Figure 4:**
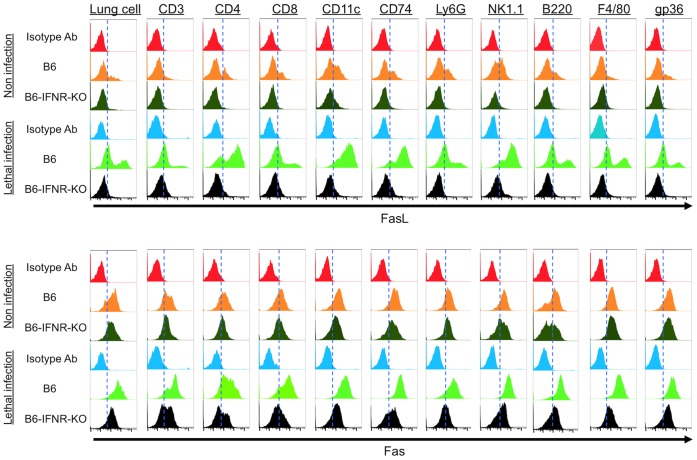
A Type-I IFN signal is essential for the induction of FasL expression on several cells in the lungs of mice lethally infected with the PR/8 virus. B6 or B6-IFNR-KO mice were infected with 10^5^ pfu/head of the PR/8 virus and sacrificed at 3DPI. The cells in the lungs isolated from the mice were stained with anti-FasL, anti-Fas, or an isotype matched control antibody (Ab) and the Abs for the indicated specific cell type marker proteins. Fluorescent activities of these samples were assessed by flowcytometry. Red or Blue color histogram shows fluorescent signal of isotype matched control Ab of the indicated cell populations in non or lethal infected condition, respectively. Orange or dark green color histogram shows that of the indicated Ab obtained from B6 or B6-IFNR-KO mice in non infected condition, and light green or black color histogram shows the signal of the indicated Ab from B6 or B6-IFNR-KO mice lethally infected, respectively. Upper panel shows results by the assay using anti-FasL specific Ab and lower shows that by the assay using anti-Fas specific Ab.

Control B6 mice or B6-IFNR-KO were infected with a lethal virus titer of the PR/8 virus (10^∧^5 pfu/head i.n.), and the expression of FasL or Fas on the cells in the lung was analyzed as described in *Materials and Methods*. In control B6 mice, protein expression of FasL was restricted to a low level in minor populations of some cell types under non-infected conditions (Fig. 4 upper panel, orange color compared with red color histogram). By lethal infection with PR/8 virus, the expression level of FasL was dramatically increased in all cell types, especially in CD4(+), CD11c(+), CD74(+) or NK1.1(+) cells (Fig. 4 upper panel, light green color compared with orange color). Contrary to these observations, the expression of FasL was not observed in all tested cell types of both non-infected and lethally infected B6-IFNR-KO mice (Fig. 4 upper panel, black or dark green color compared with light blue or red color histogram). These findings indicate that FasL expressions on the surfaces of the indicated cells were regulated by type-I IFN mediated signal. In the case of Fas protein, the expression was observed in all tested cell types in non-infected B6 control mice (Fig. 4 lower panel, orange color compared with red color histogram) and their expressions levels were slightly or not changed by lethal infection of PR/8 virus (Fig. 4 lower panel, orange color compared with light green color histogram). In non-infected B6-IFNR-KO mice, it was observed that the pattern of Fas protein expression in the indicated cell types was similar to that in non-infected B6 mice (Fig. 4 lower panel, dark green color compared with orange color histogram). It should be noted that the expression levels of Fas protein on the cells in lethally infected conditions were slightly decreased by the specific targeting of IFNR1 gene expression (Fig. 4 lower panel, black color compared with light green color histogram), but remained at a similar level in non-infected B6 control or B6-IFNR-KO mice (Fig. 4 lower panel, black color compared with dark green or orange color histogram). Therefore, these observations have prompted the suggestion that type-I IFN signal specifically associates with the change of Fas expression induced by the viral infection but not in the naïve condition. All these findings indicated that a type-I interferon signal is essential for FasL protein expression to be induced by viral infection on surface of cells in the lungs of mice.

### Difference in Time-course Kinetics of Type-I Interferon Amount in Bronchoalveolar Lavage Fluid in Lethally and Non-lethally Infected Mice

**Figure 5:**
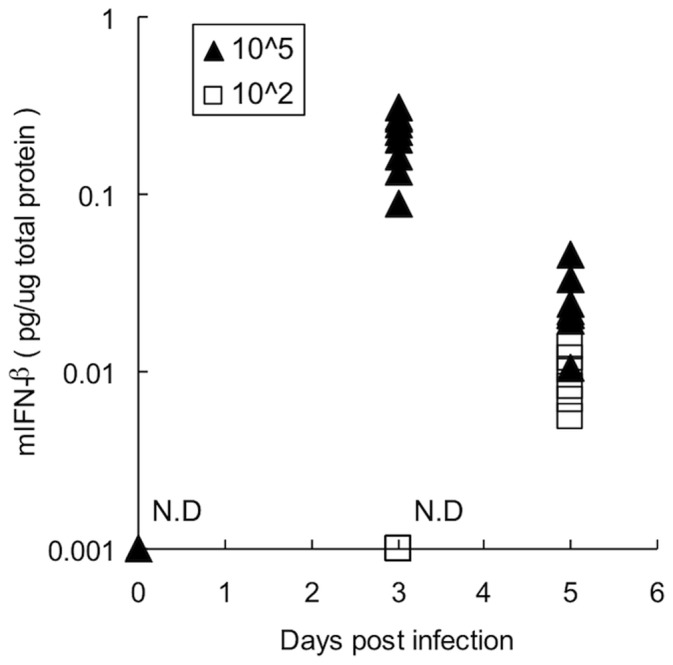
Production of IFN- ß in the lungs of mice infected lethally or non-lethally with the PR/8 virus. B6 mice were intranasally infected with 10^5^ (closed triangle) or 10^2^ (open square) pfu/head of the PR/8 virus. At 0, 3 or 5 DPI, the BALF of these mice were isolated. The amount of IFN- ß or total protein contained in these samples was assessed by mouse IFN- ß specific ELISA or BCA protein assay, respectively. The amounts of IFN- ß were normalized by that of the total protein in each sample. “N.D.” means not detected.

In the above study, it was shown that *FasL* mRNA expression in the lung of lethally infected mice was detected at earlier than in non-lethally infected mice (Fig. 3A and C). To clarify the detail of the differences in non-lethal or lethal infected conditions, the time dependent kinetics of production of type-I interferon in the lungs of mice infected non-lethally and lethally were evaluated. The amounts of type-I interferon in the broncho alveolar lavage ﬂuid (BALF) in the lungs of these mice were assessed. Murine IFN-β specific ELISA showed that production of IFN-β protein in the BALF of mice infected with a lethal titer of the PR/8 virus was induced at 3DPI and this production level was slightly decreased at 5DPI (Fig. 5). In the case of non-lethal infection, IFN-β production was not detected in the BALF at 3DPI, but was slightly detected at 5DPI (Fig. 5). These findings indicate that the time dependent kinetics of IFN-β production is different between the lethal and non-lethal infections of the virus in the lungs of mice.

## Discussion

In this study, we proposed that type-I IFN production highly induces the expression of FasL on several cells in the lung which leads to the reduction of the survival rate after a lethal infection of PR/8 virus. Previously, it was reported that intranasal administration of anti-Fas specific agonistic antibody induces acute lung inflammation [7], [8]. We also found that functional mutation of the *FasL* gene protects mice from a lethal influenza A virus infection (Fig. 1A) as well as in a previous study [6]. Our data and the previous reports suggest that FasL mediated signal in lung has a negative effect for protecting host against PR/8 virus infection. Since the same perspective was provided by the assay using the administration of a recombinant chimeric protein inhibitor for FasL/Fas interaction (Fig. 1B), this effect was not due to the other effects mediated by gld/gld mutation or genetic background before the viral infection.

In Fig. 2, it is demonstrated that the severity of illness, such as reduction of body weight and survival rate, after influenza A virus infection should correlate with the initial infected titer of the virus but not the titer of the propagated virus in the lung. In this situation, it was shown that induction of *FasL* gene in lung of mice lethally infected with PR/8 virus was detected earlier than in that of non-lethally infected mice, and this time-course kinetics seemed to correlate with loss of body weight (compared with Fig. 3A versus 3E, and Fig. 3C versus 3F). In addition, it was reported that activation of the Fas signal causes severe inflammation in the lungs of mice [7], [8]. Although the series of immunological or pathological reactions in the host are triggered by the viral infection, our findings suggest that the severity of influenza should be regulated by the host reaction associated with FasL expression, especially in the early phase of the infection. Since it was demonstrated that gld/gld mutation prevented the reduction of the survival rate (Fig. 1) but did not affect the virus titer in lung (Fig. S1), this perspective is strongly supported.

Regarding the molecular function of FasL in lung inflammation mediated by lethal infection with PR/8 virus, it is known that FasL plays an effector role in killing the virus infected cells as well as the activated lymphocytes [2]. The reduction of CD3(+) T-cell population in the lungs of mice infected with a high titer of PR/8 virus was observed and this reduction was prevented by gld/gld mutation (Fig. S2 A and B). These data and previous report [22] suggested that the FasL/Fas signal should negatively regulate the host protection system by controlling the T-cell population rather than eliminate virus-infected cells in lethal influenza virus infection.

In Fig. 4, it is demonstrated that in non-infected mice, Fas protein was expressed on several cell surfaces, but expression of FasL protein was detected on a rare population of lung cells. In B6 mice lethally infected with PR/8 virus, it was observed that expression of FasL was dramatically increased on several cell surfaces but Fas expression was not or slightly up-regulated. More importantly, this induction of FasL expression due to lethal infection was not observed in B6-IFNR-KO mice. These findings indicate that the FasL/Fas signal should be triggered by the induction of expression of FasL rather than Fas in mice infected with influenza A viruses, and this induction was regulated by type-I IFN mediated signal. Since, in the lung of control B6 mice lethally infected, higher induction of FasL expression in CD4(+), CD74(+), NK1.1(+) or CD11c(+) cells than other cell types was detected (Fig. 4, upper panel, light green color histogram), these cells should associate with the FasL mediated reduction of CD3(+) cell population in lung of mice lethally infected (Fig. S2).

As shown in above studies, there are differences in kinetics of FasL mRNA expression between lethal and non-lethal virus infections (Fig. 3 A and C). It is also demonstrated that at 3DPI, IFN- ß is largely produced after the infection with a high titer of the virus compared to that with a low titer of the virus, and their amounts are equivalent at 5DPI (Fig. 5), suggesting that FasL expression in the virus-infected mice are controlled by type-I IFN depending on its time kinetics rather than its amount. Production of type-I IFN after influenza A virus infection is regulated by two different types of viral RNA recognizing receptor proteins, such as TLRs and RIG-I like proteins. While TLRs play their essential role for production of type-I IFN in macrophages or plasmacytoid dendritic cells (DC), RIG-I like proteins are critical for their production in conventional DC or fibroblasts [12], [13]. In addition, it is proposed that in a respiratory RNA virus infection, alveolar macrophage is a main source for producing type-I IFN [23] and it is also reported that prevention of the recruitment of macrophages into the lungs protects mice against lethal PR/8 virus infection [24]. The differences in the time-kinetics of type-I IFN between the lethal and non-lethal infections might be due to the differences of mainly producing cell types such as alveolar macrophage or epithelial cells.

Type-I IFN has been identified as a virus interference agent and stimulates production of several intracellular proteins preventing virus RNA synthesis [25]. More recently, several studies proposed that type-I IFN plays important roles for inhibition of virus production as well as regulation of the activation of immune cells [14] and contributes to the progression of autoimmune disease, such as systemic lupus erythematosus [26]. These findings and our data suggest that abnormal regulation on type-I IFN production should associate with several diseases, such as viral infection and autoimmune diseases. Taken together, it is demonstrated that type-I interferon induced by the viral infection should be associated with the induction of FasL protein which should play a negative effect on host protection against lethal influenza virus infection, and it is therefore suggested to explore the detail mechanism of regulation by the FasL/Fas system for the host immunological response; doing so should be beneficial to the controlling of the severity of influenza.

## Supporting Information

Figure S1 **gld/gld mutation on** ***FasL*** **gene does not affect virus production in lung of mice lethally infected with PR/8 virus.** Control B6 or B6-gld/gld mice were infected with 10^5^ pfu/head of the PR/8 virus. At the indicated day, the mice were sacrificed and the virus titers in the isolated lungs of the mice were assessed by plaque assay as described in *Materials and Methods*.
(TIFF)

Figure S2 **gld/gld mutation on** ***FasL*** **gene prevents the reduction of CD3(+) cell population in lung of mice lethally infected with PR/8 virus. (A)** B6 (closed) or B6-gld/gld (opened) mice were infected with 10^5^ pfu/head of the PR/8 virus. At 0, 3 or 5 DPI, the mice were sacrificed and the percentages of the cell populations expressing the indicated cell type marker among live (7-AAD(−)) FSC/SSC gated lung cells were assessed by flowcytometry. (N = 3/each group). **(B)** B6 or B6-gld/gld mice were infected with 10^2^ or 10^5^ pfu/head of the PR/8 virus. At 5 DPI, the cells isolated from the lungs of these mice were analyzed as described in **A**. (N = 3/each group).
(TIFF)
