# Supplementary figures and images for: Type-I Interferon is Critical for FasL Expression on Lung Cells to Determine the Severity of Influenza
Source: PLoS One. 2013 Feb 8;8(2):e55321. doi: 10.1371/journal.pone.0055321 (PMC3568138; doi:10.1371/journal.pone.0055321)

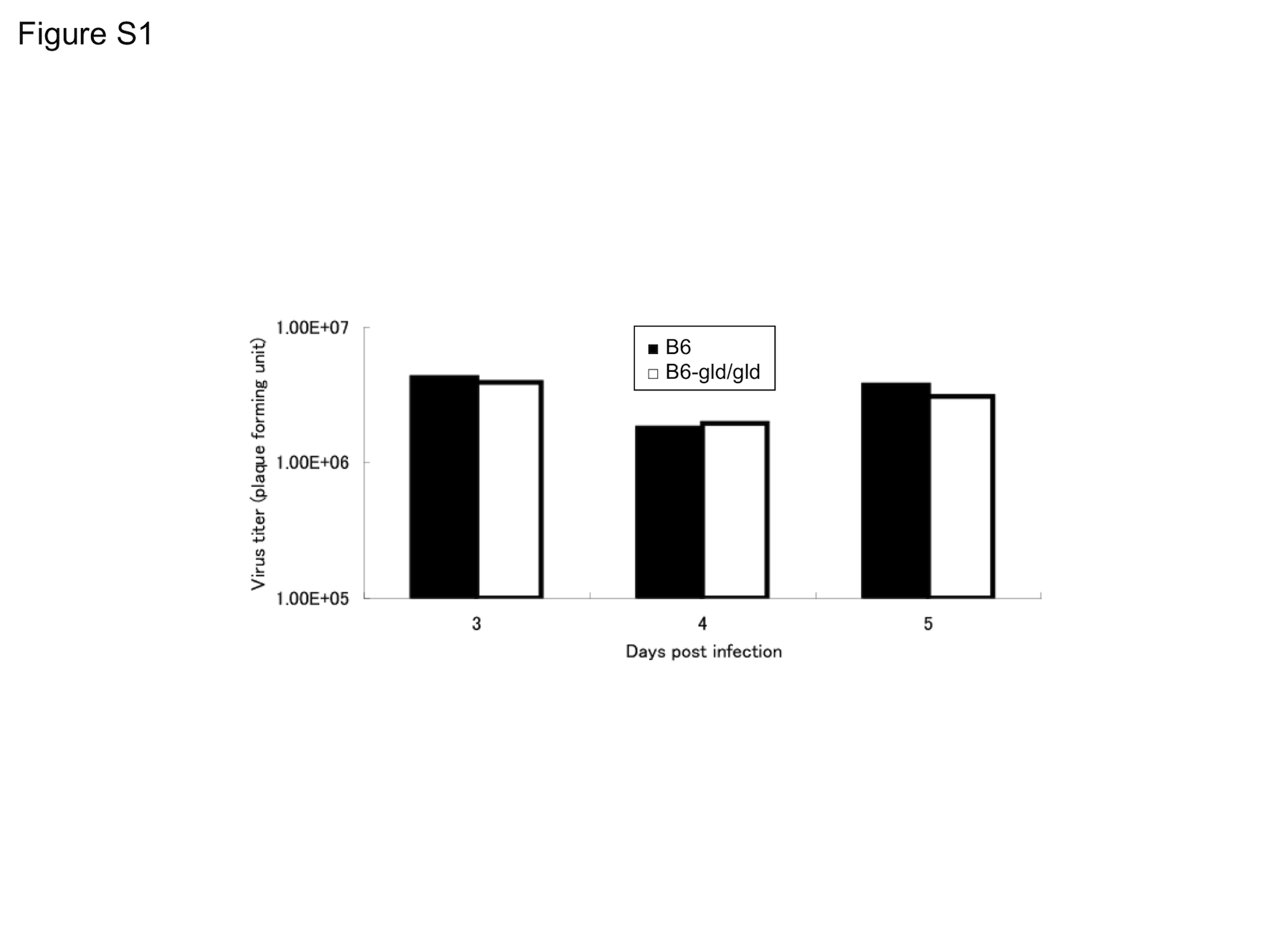

Supplement: Figure S1 — gld/gld mutation on FasL gene does not affect virus production in lung of mice lethally infected with PR/8 virus. Control B6 or B6-gld/gld mice were infected with 105 pfu/head of the PR/8 virus. At the indicated day, the mice were sacrificed and the virus titers in the isolated lungs of the mice were assessed by plaque assay as described in Materials and Methods. (TIFF) [file pone.0055321.s001.tiff]

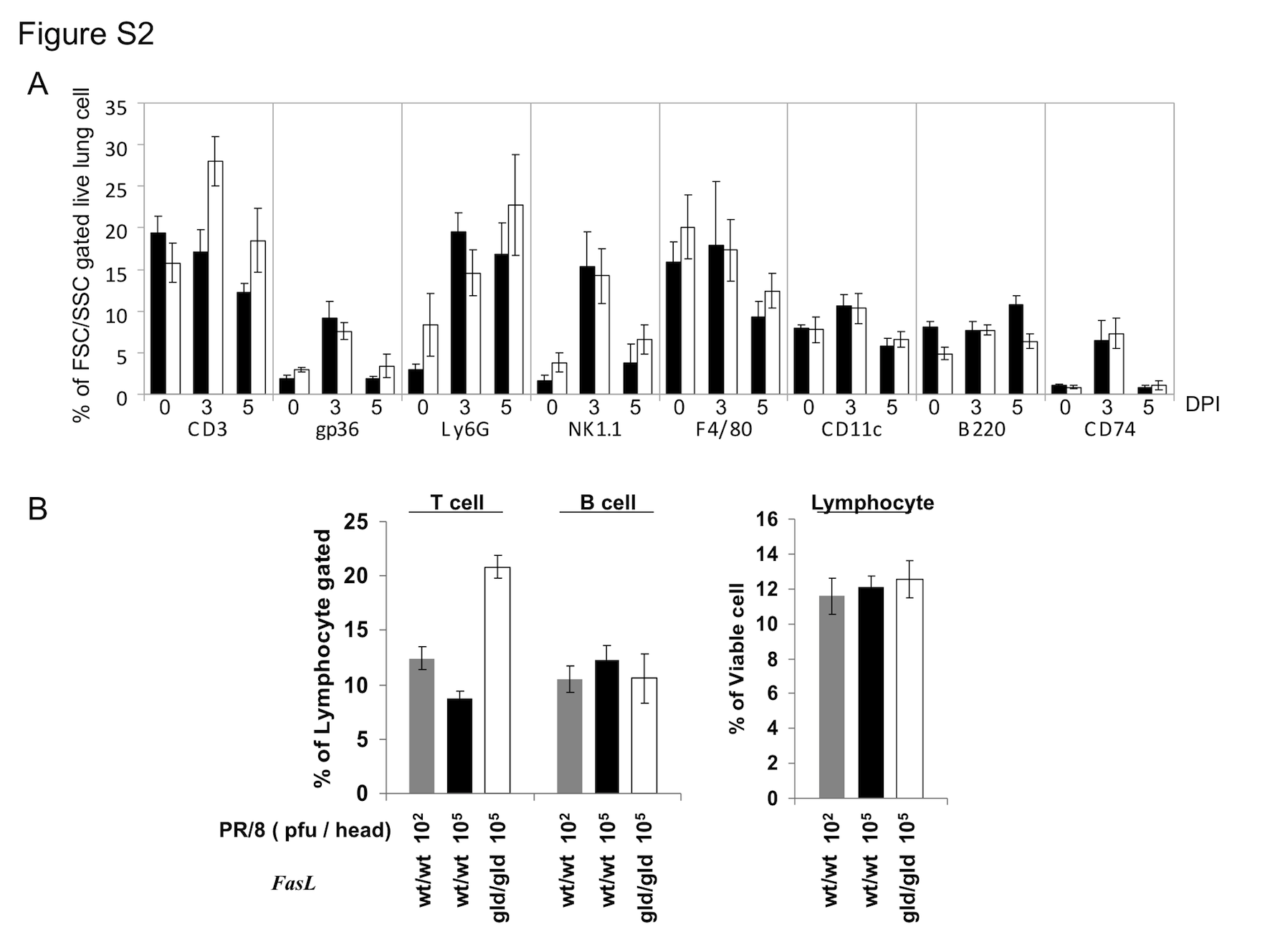

Supplement: Figure S2 — gld/gld mutation on FasL gene prevents the reduction of CD3(+) cell population in lung of mice lethally infected with PR/8 virus. (A) B6 (closed) or B6-gld/gld (opened) mice were infected with 105 pfu/head of the PR/8 virus. At 0, 3 or 5 DPI, the mice were sacrificed and the percentages of the cell populations expressing the indicated cell type marker among live (7-AAD(−)) FSC/SSC gated lung cells were assessed by flowcytometry. (N = 3/each group). (B) B6 or B6-gld/gld mice were infected with 102 or 105 pfu/head of the PR/8 virus. At 5 DPI, the cells isolated from the lungs of these mice were analyzed as described in A. (N = 3/each group). (TIFF) [file pone.0055321.s002.tiff]
